# Relationship between aqueous humor cytokine level changes and retinal vascular changes after intravitreal aflibercept for diabetic macular edema

**DOI:** 10.1038/s41598-018-35036-9

**Published:** 2018-11-08

**Authors:** Rodolfo Mastropasqua, Rossella D’Aloisio, Marta Di Nicola, Giuseppe Di Martino, Alessia Lamolinara, Luca Di Antonio, Daniele Tognetto, Lisa Toto

**Affiliations:** 1 Department of Neuroscience, Polytechnic University of Marche, Ancona, 60126 Italy; 2 Eye Clinic, Department of Medicine, Surgery and Health Sciences, University of Trieste, Trieste, 34100 Italy; 3 Department of Medical, Oral and Biotechnological Sciences, Laboratory of Biostatistics, University “G. d’Annunzio” Chieti-Pescara, Chieti, 66100 Italy; 4 School of Hygiene and Preventive Medicine, Department of Medicine and Science of Ageing, University G. d’Annunzio Chieti-Pescara, Chieti, 66100 Italy; 5 Department of Medicine and Aging Science, CeSi-Met, University “G. d’Annunzio” Chieti-Pescara, Chieti, 66100 Italy; 6 Ophthalmology Clinic, Department of Medicine and Science of Ageing, University G. d’Annunzio Chieti-Pescara, Chieti, 66100 Italy

## Abstract

The aim of this work was to investigate the changes in aqueous humor cytokine levels after intravitreal injection of aflibercept in diabetic macular edema (DME) and to evaluate the relationship between cytokines modifications and central macular thickness (CMT) and retinal/choroidal vascular changes using structural and functional optical coherence tomography (OCT). Aqueous concentrations of 38 cytokines were measured via multiplex bead assay. In addition, spectral domain OCT and OCT angiography with SSADA software (XR Avanti® AngioVue) were performed at baseline and after intravitreal injections. VEGF, IL-6, IL-5, IL-1β, Eotaxin, GRO, IL-12p40, IL-12p70, IL-1RA, Flt-3L and IP-10 showed a statistically significant decrease through the follow-up (p < 0.05; p < 0.001), while Fraktalkine and GM-CSF significantly increased (p < 0.05). Best corrected visual acuity significantly increased and CMT significantly decreased during follow-up (p < 0.001 and p = 0.013). Superficial capillary plexus and deep capillary plexus density significantly increased (p < 0.001 and p = 0.014). A positive relation was found between GRO, VEGF, Fraktalkine, IP-10, IL-12p70 aqueous humor levels and CMT (p < 0.05; p < 0.001). Aflibercept is a primary anti-VEGF treatment producing a decrease of DME due to the reduction of vascular permeability, nevertheless other inflammatory cytokines showed modification after aflibercept intravitreal injections probably related to edema modification or to an interaction of aflibercept with other inflammatory cytokines.

## Introduction

Diabetic macular edema (DME) remains one of the most widespread diabetic retinopathy (DR) complications at any stage of the disease. Its prevalence ranges from 19% to 65%^1^.

DR and DME are considered chronic inflammatory conditions with high level of pro-inflammatory molecules. Capillary leakage and subretinal fluid accumulation involve the expression of several inflammatory cytokines and growth factors, particularly of vascular endothelial growth factor (VEGF)^2^.

Several studies investigating the intraocular concentrations of cytokines have revealed the relations between vitreous or humor aqueous levels of VEGF, IL-6, IL-8, inducible protein-10 (IP-10), intercellular adhesion molecule 1 (ICAM-1) and monocyte chemotactic protein 1 (MCP-1) and DME^3–6^.

In addition, increasing inflammatory cytokines concentrations have been found to be related to more severe stages of DR^7^.

The current literature has widely demonstrated that anti-VEGF agents are an effective therapy for DME and DR progression modulating the VEGF expression^3–9^.

Many studies focused on the analysis of aqueous humor cytokines changes after anti-VEGF intravitreal treatment to better understand response and targets of therapy and prognostic factors for a successful management of the disease^10–12^.

Aflibercept is one of the primary anti-VEGF treatment for DME. It decreases retinal vascular permeability due to its strong binding with VEGF factor that plays a key role in DME pathogenesis^3–8^.

The aim of the current study was to investigate changes in aqueous cytokine levels after intravitreal injection (IVI) of aflibercept in patients suffering from DME and to evaluate the relationship between cytokines modifications and functional and anatomical parameters.

## Results

20 eyes of 20 patients affected by type 2 diabetes with non-proliferative DR (NPDR) and DME were examined.

20 eyes of 20 healthy subjects were evaluated as controls.

The demographic and clinical characteristics of the DME group are summarized in Table 1.

**Table 1:** Study Population characteristics (DME group; n = 20).

Parameter	
Gender, *n (%)*	
Male	11 (55.0)
Female	9 (45.0)
Age (yr), *mean* ± *SD*	63.4 ± 7.3
Duration of DM (yr), *mean* ± *SD*	12.8 ± 7.1
Glycosylated haemoglobin (mmol/mol), *mean* ± *SD*	7.6 ± 3.6
Visual acuity (logMAR), *mean* ± *SD*	0.5 ± 0.1
Lens status, *n (%)*	
Phakia	6 (30.0)
Pseudophakia	14 (70.0)

Abbreviations: DM = diabetes mellitus; SD = standard deviation.

### Anatomical and functional parameters

Central macular thickness (CMT) significantly decreased during the whole follow-up (p = 0.003), from 429.5 µm at baseline to 256.0 µm at 150 days postoperatively (Table 2).

**Table 2:** Anatomical and functional parameters during the follow-up.

Variable	Baseline	60 days	120 days	150 days	p-value^*^
BCVA (logMar)	0.5 (0.3–0.6)	0.4 (0.3–0.5)^**‡**^	0.3 (0.3–0.5)	0.3 (0.2–0.5)	<***0.001***
CMT (µm)	429.5 (371.0–599.0)	307.0 (275.0–365.0)	261.0 (239.0–283.0)	256.0 (203.0–280.0)	***0.003***
Foveal SCPD (µm)	27.5 (19.0–31.0)	25.5 (22.0–30.4)	25.0 (20.0–32.0)	27.4 (17.2–32.3)	*0.256*
Parafoveal SCPD (µm)	40.0 (38.0–46.0)	45.0 (42.0–47.0)	45.5 (43.0–48.0)	46.4 (44.6–49.2)	<***0.001***
Foveal DCPD (µm)	21.0 (12.0–25.0)	25.0 (20.0–26.0)^**†**^	30.0 (14.0–35.0)	26.5 (19.7–34.9)	***0.050***
Parafoveal DCPD (µm)	48.5 (43.0–51.0)	48.5 (45.0–55.0)^**†**^	51.5 (49.0–52.0)	51.8 (50.3–53.4)	***0.019***
Foveal CCD (µm)	63.0 (59.5–66.3)	63.5 (57.0–65.0)	62.0 (59.0–64.0)	66.6 (63.8–66.9)	*0.061*
Parafoveal CCD (µm)	63.0 (61.8–65.3)	63.5 (62.0–65.0)	65.0 (64.0–67.0)	66.2 (64.9–67.9)	*0.085*

^*^Friedman test evaluating variation in anatomical and functional parameter among time; ^†^p < 0.05, ^‡^p < 0.001 pairwise post-hoc analysis vsprevious measurement.

Bolded p-value were significant after FDR correction.

Abbreviations: BCVA = best corrected visual acuity, CMT = central macular thickness; SCPD = superior capillary plexus density;

DCPD = deep capillary plexus density; CCD = choriocapillaris density.

On parallel, best-corrected visual acuity (BCVA) significantly increased during 5-month follow-up (p < 0.001), particularly at 60 days post IVI compared to the previous time point. Parafoveal superficial capillary plexus density (SCPD) and deep capillary plexus density (DCPD) showed a significant increase (p < 0.001 and p = 0.019; Table 2), while choriocapillaris density (CCD) did not significantly modify during the whole study.

Two out of the 20 eyes developed posterior vitreous detachment (PVD) during follow-up, one eye after the first injection and the second eye after the third injection.

### Aqueous cytokine levels

**Figure 1:**
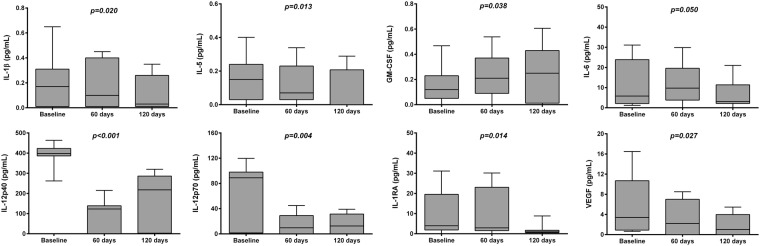
Box-whiskers graphs of Interleukins values at baseline and during the follow-up. Box-whiskers plots show the 25^th^ and 75^th^ percentile range (box) with 95% Tukey’s confidence intervals (whiskers) and median values (transverse lines in the box). The p-values in figure are relative to comparison of Interleukins levels among time.

**Figure 2:**
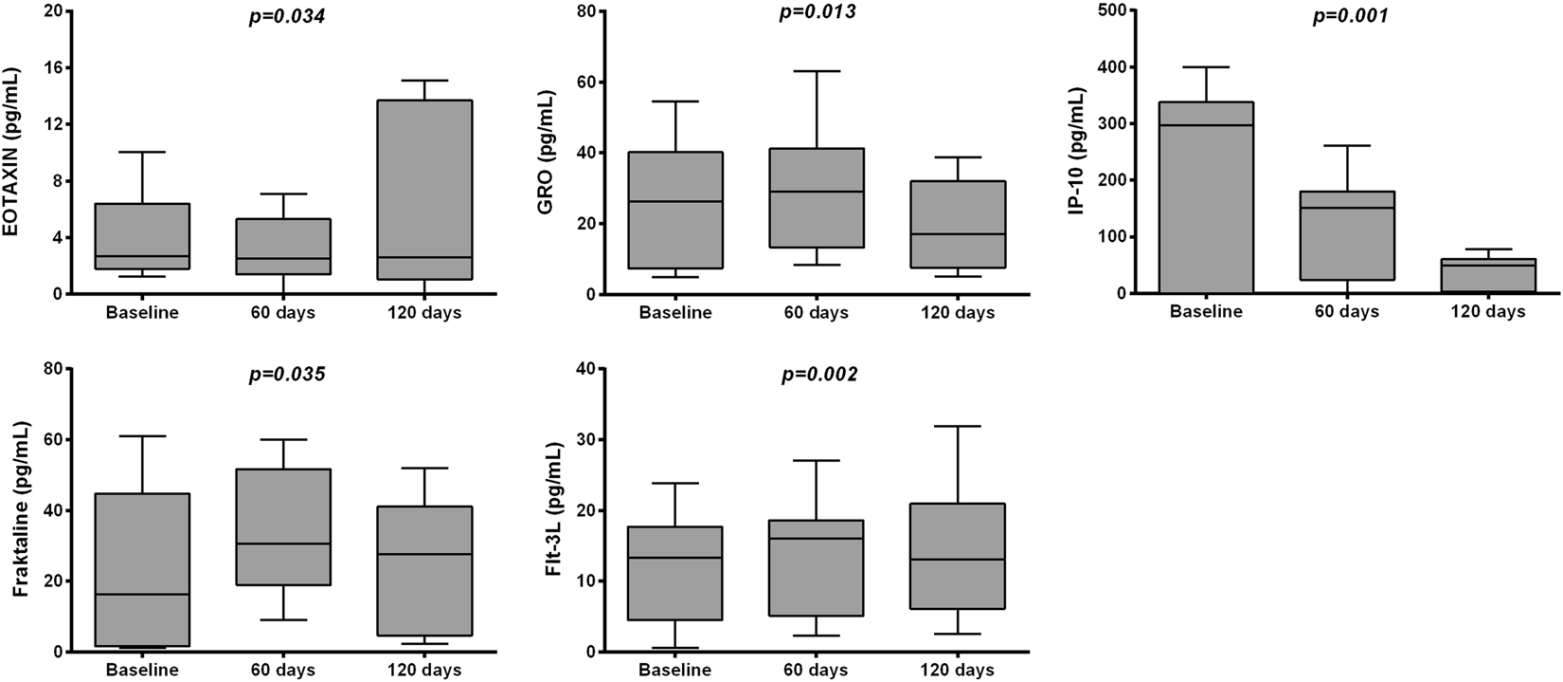
Box-whiskers graphs of Chemokines values at baseline and during the follow-up. Box-whiskers plots show the 25^th^ and 75^th^ percentile range (box) with 95% Tukey’s confidence intervals (whiskers) and median values (transverse lines in the box). The p-values in figure are relative to comparison of Chemokines levels among time.

A total of 38 cytokines were analyzed from aqueous humor samples of 20 eyes performing IVI and of 20 eyes of controls performing cataract surgery. Only those samples with measurable concentration of cytokines at baseline and at 4 weeks after the second injection and after the fourth injection were included in the analysis. Cytokine concentrations at different follow-up time are reported in Table 3, Figs 1 and 2.

**Table 3:** Aqueous cytokine levels at baseline and during the follow-up.

Cytokine (pg/mL)	Controls	Cases			Absolute variation Baseline vs 120 days	p-value^#^	p-value^*^
		Baseline	60 days	120 days			
Interleukins							
IL-1β	0.06 (0.04; 0.09)	0.17 (0.01; 0.31)	0.10 (0.01; 0.4)	0.04 (0.01; 0.26)^**†**^	−0.10 (−0.09; 0.23)	*0.147*	***0.020***
IL-1RA	3.51 (2.10; 14.35)	3.97 (1.89; 19.58)	2.94 (1.55; 23.05)	0.94 (0.54; 1.74)^**†**^	−2.16 (−4.13; −0.03)	*0.685*	***0.014***
IL-4	1.28 (0.49; 2.51)	0.57 (0.33; 2.37)	0.72 (0.33; 3.89)	0.99 (0.33; 1.65)	0.21 (−2.52; 5.49)	*0.334*	*0.917*
IL-5	0.14 (0.10; 0.41)	0.15 (0.03; 0.24)	0.07 (0.03; 0.23)^**†**^	0 (0; 0.20)	−0.16 (−0.25; −0.03)	*0.352*	***0.013***
IL-6	2.99 (1.25; 6.31)	5.84 (2.10; 24.81)	9.75 (3.81; 19.62)^**†**^	3.11 (2.15; 11.39)^**†**^	−3.05 (−6.13; 0.37)	*0.078*	***0.050***
IL-7	2.05 (1.43; 3.58)	1.86 (0.70; 4.32)	1.06 (0.70; 2.91)	0.88 (0.29; 2.54)	−0.08 (−1.99; 0.30)	*0.540*	*0.441*
IL-10	0.54 (0.16; 0.82)	1.22 (0.08; 1.99)	0.66 (0.02; 2.28)	0.41 (0; 2.16)	0 (−0.31; 0.10)	***0.005***	*0.479*
IL-12p40	3.49 (0.62; 4.78)	398.50 (386.76; 423.30)	123.48 (0.74; 139.78)^**†**^	218.01 (2.79; 286.01)^**†**^	−196.87 (−396.94; 132.45)	<***0.001***	<***0.001***
IL-12p70	2.05 (0.34; 2.39)	89.52 (1.62; 98.35)	9.73 (0.34; 29.21)^**‡**^	12.59 (0.34; 31.35)	−81.91 (−98.36; 7.59)	<***0.001***	***0.004***
IL-15	1.91 (0.87; 3.59)	4.62 (3.17; 9.88)	2.69 (1.95; 8.91)	5.33 (2.52; 11.51)	1.94 (−4.78; 1.19)	<***0.001***	*0.097*
G-CSF	1.11 (0.74; 1.87)	1.23 (0.74; 4.03)	1.40 (0.42; 3.46)	1.16 (0.06; 2.02)	−0.88 (−3.89; 0.42)	*0.740*	*0.301*
GM-CSF	0.27 (0.13; 0.54)	0.12 (0.05; 0.23)	0.21 (0.09; 0.37)^**‡**^	0.25 (0.03; 0.43)	0.14 (−0.1; 0.25)	*0.350*	***0.038***
GrowthFactors							
EGF	1.48 (0.25; 6.50)	3.28 (1.23; 4.48)	0.88 (0.25; 3.3)	1.26 (0.25; 3.02)	−4.55 (−15.83; −1.28)	***0.047***	*0.067*
FGF-2	20.48 (11.65; 395.00)	5.42 (0.28; 8.41)	5.15 (3.50; 12.23)	3.55 (3.50; 12.04)	−1.78 (−2.35; 0.11)	***0.001***	*0.717*
VEGF	0.43 (0.12; 0.88)	3.41 (0.91; 10.75)	2.19 (0.40; 7.05)^**†**^	1.03 (0.40; 3.98)	−0.09 (−4.22; 2.48)	<***0.001***	***0.027***
Interferons							
IFNα2	1.11 (0.86; 5.10)	2.65 (0.18; 8.16)	2.84 (0.18; 7.75)	3.87 (1.07; 4.71)	0.33 (−3.18; 1.41)	*0.531*	*0.457*
Chemokines							
MCP-1	707.00 (435.34; 1891.00)	789.00 (0.97; 1399.75)	1262.50 (692.50; 2315.50)	1088.50 (151.00; 3004.00)	−78.71 (−282.32; 618.84)	*0.451*	*0.670*
MIP-1α	1.69 (1.69; 2.61)	3.96 (1.34; 8.95)	3.47 (0.65; 6.94)	3.56 (1.58; 6.82)	−0.40 (−1.92; 1.72)	*0.238*	*0.273*
MIP-1β	4.77 (1.75; 6.69)	6.48 (1.39; 11.86)	3.53 (0.70; 7.14)	3.23 (0.55; 5.92)	−6.92 (−7.81; −4.00)	***0.015***	*0.441*
MCP-3	1.55 (1.06; 3.29)	2.53 (0.65; 4.54)	1.7 (0.16; 4.08)	0.65 (0.06; 2.36)	−3.54 (−5.39; −1.31)	***0.003***	*0.078*
EOTAXIN	0.53 (0.42; 5.40)	2.71 (1.79; 6.4)	2.54 (1.40; 5.32)^**†**^	2.61 (1.06; 13.68)	−2.67 (−4.67; 1.53)	*0.072*	***0.034***
MDC	1.43 (1.43; 3.25)	15.56 (4.50; 20.55)	8.75 (3.49; 14.67)	2.68 (1.43; 3.69)	−14.71 (−19.84; −3.59)	***0.007***	*0.068*
GRO	21.35 (6.98; 39.31)	26.35 (7.48; 40.21)	29.08 (13.36; 41.23)	17.08 (7.60; 31.88)^**‡**^	−12.21 (−21.65; 2.72)	*0.896*	***0.013***
IL-8	4.96 (1.86; 10.12)	4.41 (1.86; 10.63)	7.83 (3.00; 27.53)	7.18 (2.41; 22.98)	−1.95 (−6.00; 6.60)	*0.370*	*0.264*
IP-10	166.00 (38.89; 236.00)	292.97 (0.31; 338.50)	151.68 (24.12; 180.10)^**‡**^	48.93 (3.37; 60.75)	−175.60 (−300.71; 0)	*0.636*	***0.001***
Fractalkine	8.68 (5.05; 14.33)	16.3 (1.61; 44.88)	30.55 (18.90; 51.76)^**†**^	27.77 (4.61; 41.21)	2.28 (−4.14; 9.48)	*0.494*	***0.035***
Flt_3L	12.37 (2.73; 19.75)	13.31 (4.23; 17.78)	16.01 (5.07; 18.59)^**‡**^	13.14 (6.10; 20.95)	−0.21 (−12.40; 4.81)	*0.764*	***0.002***

Cytokine levels are expressed as median and Interquartile range (IQR).

^*^Friedman test evaluating variation in cytokine levels among time; ^†^p < 0.05, ^‡^p < 0.01 pairwise post-hoc analysis vs previous.

measurement.

Bolded p-value were significant after FDR correction.

^#^Baseline vs controls.

For same cytokines, the concentration values were at the lowest test sensitivity threshold; therefore exact concentration values are not presented. The excluded cytokines were IL-1α, IL-2, IL-9, TGFα, IFNγ, sCD40L and TNFα.

A statistically significant difference between controls and subjects with diabetes at baseline was observed in aqueous humor levels of the following cytokines: IL-10, IL-12p70, IL-12p40, IL-15, EGF, FGF-2, VEGF, MIP-1β, MCP-3 and MDC (p < 0.05; p < 0.001; Table 3).

VEGF levels showed a significant reduction from baseline to the last postoperative control (120 days) (p = 0.027; Table 3).

Other cytokines such as, IL-6, IL-5, IL-1β, Eotaxin, GRO, IL-12p70, IL-12p40, IP-10, Flt-3L and IL-1RA showed a statistically significant decrease through the follow-up (p < 0.05; p < 0.001), while Fraktalkine and GM-CSF significantly increased (p < 0.05; Table 3).

A positive and significant relationship was found between absolute variation of GRO, VEGF, Fraktalkine, IP-10, IL-12p70 aqueous humor levels at 120 days and absolute variation of CMT (p < 0.05; p < 0.001; Table 4). A negative and significant relationship was found between absolute variation of VEGF aqueous humor levels and absolute variation of BCVA (p = 0.041; Table 4), while a positive and significant relationship between absolute variation of IL-1RA and BCVA was detected (p = 0.025; Table 4).

**Table 4:** Univariate linear mixed model analyses between the absolute variation of aqueous cytokines levels and anatomical and functional parameters.

Absolute variation of cytokine levels (pg/mL)	Absolute variation of anatomical and functional parameters					
	BCVA (logMAR)		CMT (µm)		Parafoveal SCPD (µm)	
	b(SE)	*p-value*	b(SE)	*p-value*	b(SE)	*p-value*
IL-1β	−0.250 (0.21)	*0.239*	0.505 (0.34)	***0.136***	**−0.890** (**0.08)**	***0.001***
IL-1RA	**0.455** (**0.19)**	***0.025***	0.526 (0.26)	***0.118***	−0.127 (0.14)	***0.726***
IL-5	0.209 (0.10)	*0.326*	0.207 (0.26)	***0.567***	**0.760** (**0.21)**	***0.010***
IL-6	−0.244 (0.21)	*0.251*	−0.177 (0.56)	***0.624***	**0.483** (**0.19)**	***0.017***
IL-12p40	0.140 (0.18)	*0.515*	0.065 (0.27)	***0.858***	0.146 (0.05)	***0.686***
IL-12p70	0.169 (0.22)	*0.430*	**0.729** (**0.21)**	***0.017***	−0.055 (0.14)	***0.880***
VEGF	**−0.420** (**0.20)**	***0.041***	**0.637** (**0.31)**	***0.047***	**−0.729** (**0.10)**	***0.017***
EOTAXIN	0.305 (0.10)	*0.147*	0.383 (0.22)	***0.274***	−0.395 (0.17)	***0.258***
GRO	−0.105 (0.21)	*0.625*	**0.809** (**0.25)**	***0.005***	−0.298 (0.18)	***0.402***
IP-10	−0.048 (0.28)	*0.824*	**0.718** (**0.21)**	***0.019***	**−0.940** (**0.30)**	<***0.001***
Fracktalkine	−0.143 (0.22)	*0.505*	**0.900** (**0.66)**	<***0.001***	−0.529 (0.58)	***0.116***
FLT-3L	0.027 (0.01)	*0.901*	0.449 (0.39)	***0.193***	0.069 (0.14)	***0.849***

Abbreviations: BCVA = best corrected visual acuity, CMT = central macular thickness; SCPD = superior capillary plexus density; b = regression coefficient adjusted for age, gender and duration of diabetes; SE = standard error.

Bolded p-value were significant after FDR correction.

No statistically significant relationships were found between absolute variation of cytokine levels and absolute variation of foveal and parafoveal DCPD (data not shown).

Aqueous levels of VEGF, IL-1β and IP-10 were significantly and negatively related to parafoveal SCPD (p < 0.05, p < 0.001; Table 4). On the contrary, IL-6 and IL-5 were significantly and positively related to parafoveal SCPD (p < 0.05; Table 4).

## Discussion

Aflibercept is considered a first-line therapy for central-involved DME and has been demonstrated to have a higher gain in visual acuity at 1-year follow-up than bevacizumab and ranibizumab^13^.

It specifically targets retinal endothelial cells (REC) decreasing their permeability due to its blockage effect on VEGF-A. The latter has been reported to be higher in DR patients at all stages^14^. However, some patients do not respond to anti-VEGF treatment suggesting that other inflammatory factors are probably involved in DME pathogenesis^15^.

The current study evaluated the influence of anti-angiogenic therapy using aflibercept on different cytokines in the aqueous humor of DME patients.

VEGF and several inflammatory cytokines have been related to DME development, although the real mechanism of each molecule is still unclear^15^. Several cytokines showed modifications after IVI of anti-VEGF, probably related to interaction of the drug with other possible contributors to DME. The VEGF-induced effects are supposed to be influenced and mediated by other cytokines with a cascade mechanism^15^.

It has already been reported that *in vitro* VEGF-induced REC proliferation can be prevented from anti-VEGF therapies. Aflibercept can also bind placental growth factor (PIGF) as well, conversely to bevacizumab and ranibizumab, with a higher capability to inhibit proliferation and migration processes of REC. Moreover, the restoration of REC barrier was obtained with lower concentrations of aflibercept compared to ranibizumab^4^.

Funatsu *et al*.^4^ reported that VEGF, ICAM-1, IL-6 and MCP-1 in the vitreous fluid were statistically significantly higher in patients suffering from diabetes than those without diabetes and found higher levels in severe DME, defined as hyperfluorescent, than in mild fluorescent DME characterized by a reduced fluorescein leakage at the macula.

In addition, all these molecules showed a significant overall correlation with CMT but only VEGF and ICAM-1 were significant associated with the level of DME severity.

Aqueous levels of VEGF have been previously reported to be significantly correlated with IL-6 concentration in aqueous humor^4^. IL-6 is an inflammatory cytokine involved in enhancement of vascular permeability in DME. Aqueous levels of IL-6 have been found significantly higher than those in plasma^4^. However, it has not been found any significant association between IL-6 and severity of DME^4^.

In our study, VEGF showed a significant reduction after aflibercept treatment with decreasing values after subsequent injections. Our data described a significant reduction of IL-6 after treatment and no relationship between IL-6 and CMT as well. Moreover, a significant relationship between IL-6 and SCPD in the parafoveal area was reported supporting its role in retinal microvascular damages^4^.

Higher level of inflammation-induced molecules such as IP-10 and MCP-1 were observed in subjects with diabetic maculopathy^16^.

IP-10 is a chemokine secreted by monocytes, endothelial cells and fibroblasts, which enhances the T-helper type 1 immune reactivity and has been reported to inhibit angiogenesis^17^. IP-10 has been positively related with increased VEGF levels in patients with DR^18^.

MCP-1 has been identified as an inducer of endothelial cell chemotaxis *in vitro* and as a mediator of inflammatory angiogenesis *in vivo*^16^.

A significant decrease of IP-10 and MCP-1 were not found after anti-VEGF injections, but only after corticosteroids as Yu’s *et al*. reported, suggesting different targets and pharmacokinetics of the two therapies^16^.

On the contrary, Shiraya *et al*. found significant reduction of IP-10 after ranibizumab intravitreal injection for DME, correlating with the decrease of central retinal thickness^19^.

In our study IP-10 decreased significantly during follow-up and was positively related to CMT.

IL-1β is a pro-inflammatory and pro-neovascularization molecule that has previously been described to be significantly involved in retinal microvascular damages of diabetic maculopathy disease^20^. A significant reduction of IL-1β during the treatment period was found in our sample.

Moreover, our study reported significant changes of Eotaxin and GM-CSF levels in aqueous humor after anti-VEGF therapy. The reduction of Eotaxin concentration has already been observed in Shiraya *et al*.’s^19^ study after ranibizumab injections. Lower levels of Eotaxin have been considered as a prognostic factor for therapy response^19^.

GRO is a chemokine belonging to the CXC family, which recruits neutrophils and basophils and is involved in the action of inflammation and angiogenesis^21^.

Increased values of GRO level were found in the plasma of patients with diabetes compared to the controls and in the vitreous of patients with proliferative diabetic retinopathy (PDR)^22^.

In our study GRO significantly reduced during treatment and was directly related to CMT.

Fractalkine, the sole member of the CX3C chemokine family, is an angiogenic mediator *in vitro* and *in vivo* and has been found elevated in patients with PDR^23^.

In our study Fractalkine was significantly related to CMT, but significantly increased during follow-up after repeated aflibercept injections. The significance of this result should be better explored.

IL-5 is a B-cell–proliferating factor. Concentration of IL-5 has been reported higher in DR patients compared to patients suffering from diabetes without retinopathy and among different stages of DR IL-5 has been found higher in PDR than NPDR^24^. This finding has suggested a role of IL-5 in the development of retinal neovascularization in patients with DR. In our cases the IL-5 resulted reduced after treatment with continuous decrease after repeated injections. This is in agreement with previous studies that demonstrated a reduction of IL-5 after IVI of anti VEGF, such as ranibizumab and bevacizumab related to reduction of retinal thickness^25^.

IL-12p40 has been found increased in PDR patients compared to normal controls and has been hypothesized to have a role in the inhibition of retinal neovascularization^26^.

In our study IL-12p40 continuously decreased during follow-up after aflibercept injections.

It has been shown that intraocular cytokine concentration may change after intravitreal injection if a posterior vitreous detachment occurs^27,28^.

Our series showed the occurrence of posterior vitreous detachment in 2 out of 20 eyes during follow-up that could have partially caused the detection of lower levels of cytokines concentration.

In our work retinal vessel density changes were also studied to analyze the action of aflibercept on anatomical parameters of the patients. A significant increase of superficial and deep vessel density was observed during follow-up.

Retinal capillary vessels increase in the superficial and deep plexuses was probably due to a vascular rearrangement after edema reduction or disappearance as shown by central retinal thickness decrease.

A contribution to vessel density changes was also probably due to vessel caliber changes related to anti-VEGF treatment^29^. Recently, a prospective cohort study reported the significant association between the level of VEGF at baseline and the anatomic response in terms of center-involving DME improvement after the intravitreal treatment^12^.

Correlations analyses between cytokines and vessel density showed that reduction of aqueous levels of VEGF, IL-1β and IP-10 during follow-up was significantly related to an increase of parafoveal superficial vessel density possibly due to a reduction of macular edema after anti-VEGF treatment or to a direct action of anti VEGF on vessel caliber.

On the contrary, the reduction of aqueous level of IL-6 and IL-5 during follow-up were significantly related to a reduction of superficial parafoveal vessel density. The latter result is not easily understandable considering the reduction of macular edema; it can be hypothesized that an interaction among cytokines and particularly between these two cytokines and VEGF could lead to a direct action on vessel caliber causing variation of vessel density.

During repeated injections there were cytokines (VEGF, IL-5, IL-1β, Eotaxin, IL-12p70, IL-12p40, IP-10 and IL-1RA) decreasing after the first injections (first 2 injections) that continued to decrease significantly through the follow-up, cytokines (IL-6, GRO) that slightly increased after the first 2 injections and decreased significantly thereafter through the follow-up compared to baseline values, while other cytokines (Fraktalkine and GM-CSF) increasing after the first 2 injections that significantly increased with subsequent injections. The behavior of aqueous humor cytokines was consistent with the continuous central retinal thickness reduction and retinal vessel density increase during follow-up.

Roh *et al*. demonstrated the continuous VEGF reduction after consecutive anti-VEGF injections in age related macular degeneration that paralleled the anatomical and functional changes^30^.

The association between continuous variation of cytokines and continuous anatomical variation during treatment confirms the efficacy of repeated injections in retinal pathologies such as DME and seems to be essential to prevent possible relapses.

This study has some limits, such as the small sample size and a short follow-up suggesting that a wider period would be needed to investigate long term changes of cytokines in aqueous of patients suffering from DME and to better understand the timing of therapy efficacy. In addition, this work did not investigate samples of proliferative diabetic retinopathy cases but restricted the analysis only in patients with NPDR and DME.

In conclusion our study showed that in DME patients morphological parameters such as retinal thickness and retinal vessel density significantly improved after aflibercept injections during a 5-month follow-up period. Functional improvement in terms of BCVA increase was also observed during follow-up, although visual acuity improvement did not reflect the anatomical improvement in terms of central macular thickness at the end of follow-up. This could be probably related to the morphological status of the central retina. It has been found that integrity of the ellipsoid band and the preservation of the external limiting membrane are predictors of functional improvement after treatment^31^.

In addition, we found through the follow-up a significant decrease of some cytokines levels (VEGF, IL-6, IL-5, IL-1β, Eotaxin, GRO, IL-12p70, IL-12p40, IP-10, Flt-3L and IL-1RA) and the increase of other cytokines (Fraktalkine and GM-CSF).

Of interest among different correlations between cytokines and anatomical and functional results was the positive relationship between absolute variation of VEGF and absolute variation of central macular thickness and the negative relationship between absolute variation of VEGF levels and absolute variation of BCVA. Moreover, superficial vessel density was significantly inversely related to VEGF.

## Methods

### Subjects and design

Twenty eyes of 20 type 2 diabetes mellitus and diabetic retinopathy patients with NPDR, according to the simplified version of the ETDRS classification and complicated by macular edema (DME group) (11 males; 9 females; mean age of 63.4 ± 7.3 years) were enrolled in the study. If both eyes of a patient met the inclusion/exclusion criteria, the eye with higher CMT was selected as the study eye. Twenty eyes of 20 healthy subjects, without any ocular disease except for cataract, and about to undergo cataract surgery were considered as controls (control group) (12 males; 8females; mean age of 60.9 ± 7.3 years). The enrollment period of both two groups was between June 2016 and January 2017 at the University “G. d’Annunzio”, Chieti-Pescara, Italy. All patients of DME group were treatment naïve. They underwent 5 consecutive IVI of 2 mg aflibercept (Eylea, Regeneron Pharmaceuticals) 30 days apart from each other.

All subjects enrolled in the study were diagnosed assessing DR using color fundus photography, fluorescein angiography (FA), spectral domain optical coherence tomography (SD-OCT) and were evaluated with a comprehensive ophthalmologic examination. This prospective study adhered to the tenets of the Declaration of Helsinki and was approved by the Ethics Committee “Department of Medicine and Science of Aging, University “G. D’Annunzio” Chieti-Pescara, Italy” (LED, n° 05, March 2016). Written informed consent was obtained from the subjects after explanation of the nature and possible consequences of the study.

Criteria for inclusion were: (1) age >18 years old; (2) BCVA greater than 0.5 LogMAR in the study eye at baseline examination; (3) presence of recent DME; (4) CMT > 300 µm as measured using the SD-OCT at the baseline examination.

The exclusion criteria were: (1) any previous ocular surgery (included IVI) in the last 6 months; (2) laser treatments; (3) retinal vascular diseases; (4) vitreo-retinal interface diseases; (5) inflammatory eye diseases; (6) medium lens opacities (according to Lens Opacities Classification System); (7) glaucoma or ocular hypertension.

### SD-OCT Angiography with XR Avanti and vascular layer segmentation

SD-OCT (XR Avanti®; Optovue, Inc., Fremont, CA, USA) and OCT angiography (OCTA) with SSADA software (XR Avanti® AngioVue) were performed in all the participants at baseline and 4 weeks after each injection.OCTA scans were acquired following a standardized protocol based on the SSADA algorithm (version 2017.1.0.144) as previously described^32^. Vascular retinal layers were visualized and segmented, as previously described, in the superficial capillary plexus (SCP), deep capillary plexus (DCP) and choriocapillaris (CC)^33^.

### Quantitative vessel analysis

Objective quantification of vessel density was carried out for each eye using SSADA software. A quantitative analysis was performed on the OCTA en-face images for each eye using AngioVue software as previously described^32^.

### Surgical procedure and sample collection

DME group underwent an IVI of 2 mg (0.05 mL) of aflibercept using the standard injection procedurein the operating room. For each patient an aqueous sample was collected at baseline before the first injection (T0), at 60 days (T1) before the third IVI and at 120 days (T2) before the fifth IVI and during cataract surgery procedure for control group, by aspirating 0.05–0.1 mL of aqueous using a sterile syringe with a 30-gauge needle at the temporal limbus. Aqueous humor samples were rapidly frozen at −80 °C until assayed.

### Aqueous humor sampling and analysis of cytokines

Aqueous humor samples were used to quantify the production of 38 cytokines using Milliplex Human Cytokine/Chemokine Magnetic Bead Panel (HCYTMAG-60K-PX38, Millipore, Billerica, MA) according to manufacturer’ protocols. This approach allowed for the simultaneous measurement of the following human analytes: EGF, FGF-2, Eotaxin, TGF-α, G-CSF, Flt-3L, GM-CSF, Fractalkine, IFNα2, IFNγ, GRO, IL-10, MCP-3, IL-12p40, MDC, IL-12p70, IL-13, IL-15, sCD40L, IL-17A, IL-1RA, IL-1α, IL-9, IL-1β, IL-2, IL-3, IL-4, IL-5, IL-6, IL-7, IL-8, IP-10, MCP-1, MIP-1α, MIP-1β, TNFα, TNFβ, VEGF. Briefly, undiluted aqueous humor samples (25 µl neat per well) were thawed and mixed well by vortexing prior to add to 25 μl of Assay Buffer. Then, 25 μl of magnetic beads coated with specific antibodies were added to this solution and incubated overnight at 4 °C with shaking. At the end of the incubation, the plate was washed twice with Wash Buffer and incubated 1 hour with 25 μl of biotinylated Detector Antibody at RT. Then, the plate was incubated for 30 minutes with Streptavidin–Phycoerythrin at RT, washed twice, and incubated with 150 μl of Sheath Fluid for 5 minutes at RT. The plate was ran immediately on a Luminex® 100™/200™ platform (Luminex Corporation, Austin, TX) with xPONENT 3.1 software. Standard curves for each analyte (in duplicate) were generated by using the reference standards supplied with the kit. Analytes concentrations in sample were determined with a 5-parameter logistic curve. Final concentrations were calculated from the mean fluorescence intensity and expressed in pg/mL. The assay was performed in a 96-well plate, using all the assay components provided in the kit. All incubation steps were performed at room temperature and in the dark to protect the beads from light.

When biochemical variables had sample values below the lower limit of detection (Out-Of-Range values or OOR); when OOR values were over the 30% of the total sample, the analytes were excluded.

### Main outcome measures

BCVA; CMT; SCPD, DCPD and CCD in the foveal and parafoveal area were evaluated at baseline and 30 days after each IVI for a total follow-up period of 150 days in all patients. Aqueous humor levels of 38 cytokines were analysed at baseline (before the first IVI), and at 60 days (before the third IVI) and at 120 days (before the fifth IVI) for the DME group and during cataract surgery procedure for the control group.

Relationship between variation of morphological, functional parameters and aqueous humor cytokines levels were evaluated in all patients.

### Statistical analysis

A Shapiro-Wilk’s test was performed to evaluate the departures from normality distribution for each variable. Mann-Whitney’s U test was performed to evaluate differences between controls and cases cytokines levels at baseline. Non-parametric Friedman test was performed to evaluate differences of each parameter over the time from baseline to 150 days measurements. Pairwise post-hoc analysis was then performed using a Wilcoxon-Nemenyi-McDonald-Thompson symmetry test.

The relationships among absolute variation of aqueous cytokines levels and absolute variation of morphological and functional parameters that are significantly at univariate analysis were estimated by linear mixed regression models, adjusted for age, gender and history of diabetes.

The number of type I errors, was controlled applying the Gavrilov-Benjamini-Sarkar procedure to bound the false discovery rate (FDR) ≤ 0.05.

Statistical analysis was performed using IBM^®^ SPSS Statistics v 20.0 software (SPSS Inc, Chicago, Illinois, USA).
